# Right ventricular phenotype, function, and failure: a journey from evolution to clinics

**DOI:** 10.1007/s10741-020-09982-4

**Published:** 2020-06-17

**Authors:** Yannick J. H. J. Taverne, Amir Sadeghi, Beatrijs Bartelds, Ad J. J. C. Bogers, Daphne Merkus

**Affiliations:** 1 Department of Cardiothoracic Surgery, Erasmus University Medical Center, Room Rg627, Dr. Molewaterplein 40, 3015 GD Rotterdam, The Netherlands; 2 Division of Experimental Cardiology, Department of Cardiology, Erasmus University Medical Center, Rotterdam, The Netherlands; 3 Unit for Cardiac Morphology and Translational Electrophysiology, Erasmus University Medical Center, Rotterdam, The Netherlands; 4 Division of Pediatrics, Erasmus University Medical Center, Rotterdam, The Netherlands

**Keywords:** Functional anatomy, Evolutionary proxies, Specific right ventricular biomechanics, Adoptive alterations

## Abstract

The right ventricle has long been perceived as the “low pressure bystander” of the left ventricle. Although the structure consists of, at first glance, the same cardiomyocytes as the left ventricle, it is in fact derived from a different set of precursor cells and has a complex three-dimensional anatomy and a very distinct contraction pattern. Mechanisms of right ventricular failure, its detection and follow-up, and more specific different responses to pressure versus volume overload are still incompletely understood. In order to fully comprehend right ventricular form and function, evolutionary biological entities that have led to the specifics of right ventricular physiology and morphology need to be addressed. Processes responsible for cardiac formation are based on very ancient cardiac lineages and within the first few weeks of fetal life, the human heart seems to repeat cardiac evolution. Furthermore, it appears that most cardiogenic signal pathways (if not all) act in combination with tissue-specific transcriptional cofactors to exert inductive responses reflecting an important expansion of ancestral regulatory genes throughout evolution and eventually cardiac complexity. Such molecular entities result in specific biomechanics of the RV that differs from that of the left ventricle. It is clear that sole descriptions of right ventricular contraction patterns (and LV contraction patterns for that matter) are futile and need to be addressed into a bigger multilayer three-dimensional picture. Therefore, we aim to present a complete picture from evolution, formation, and clinical presentation of right ventricular (mal)adaptation and failure on a molecular, cellular, biomechanical, and (patho)anatomical basis.

## Introduction

The right ventricle (RV) has long been perceived as the “low-pressure bystander” of the left ventricle (LV). The lack of interest in RV pathobiology was primarily based on early studies that showed practically no increase in venous pressure when damaging the RV free wall [1, 2] and hence underestimated RV function as a potential player in biventricular function [3]. For a long period of time, the Fontan procedure, in which the absence of a sub-pulmonary ventricle had (within certain limits) negligible effects on overall heart function [4], supported a very limited role for the RV. Consequently, extensive research on RV form and function has been scarce. In most cases, the functionality of the RV was merely extrapolated from studies based on LV function.

Only recently was the RV “rediscovered” due to evidence indicating that the RV can indeed be a significant contributor to functional hemodynamics and cardiac output [5–7], particularly in the setting of pulmonary hypertension (PAH) and congenital heart disease (CHD). Importantly, new insights show that although the RV consists of, at first glance, the same cardiomyocytes as the LV, it is in fact derived from a different set of precursor cells, with complex three-dimensional anatomy and a very distinct contraction pattern [8]. Recent studies further sparked this renewed interest, showing that RV function is an important predictor of survival in patients with heart failure (HF), PAH, and CHD [9–11]. Nevertheless, profound knowledge of normal loading conditions and physiological adaptations ensuing morphology of the RV remains limited [12–15]. Furthermore, a significant caveat exists on the difference between pressure and volume loading of the RV, as most of the current research is performed in a model of PAH or pulmonary artery banding [15].

The mechanisms of RV failure, its detection and follow-up, and more specifically the different responses of the RV to pressure versus volume overload are still incompletely understood. Despite many apparent similarities between the LV and RV, there are significant differences on a morphological, physiological, and molecular level, evidenced by different responses of the ventricles to current HF therapies [6, 16–22]. Notably, RV failure is not an entity on itself but rather a continuum of clinical symptoms related to increased severities of disease states [3, 6, 17] and associated with significant morbidity and mortality both in patients with HF with reduced [23, 24] and preserved [25, 26] ejection fraction (HFpEF). This raises the question of whether there is a “point of no return” within the pathobiological continuum of RV failure and if RV dysfunction is a marker of severity of HF or a separate entity leading to the worse prognosis of HF. Despite a lot of new research on RV physiology and clinical manifestations of RV failure, a translational approach to comprehend all aspects, linking morphology to function, is still lacking. Within this review, we aim to provide an overview of the developmental biology of the RV with a focus on the relationship between form and function. More specifically, current visions on the different cellular and molecular pathways and biomechanical implications on the spectrum from RV function to adaptation and failure will be discussed.

## Evolutionary biology and embryology of the right ventricle

### Comparative anatomy of the heart

In order to fully comprehend the functional anatomy of the RV and the intricate relation between form and function, we need to explore the evolutionary pathway leading to the formation of the RV. Indeed, studying common precursors could provide us with additional information on the formation and possible maladaptation of the RV dealing with altered loading conditions [27]. When comparing all vertebrate species, no fundamental differences exist in the phenotype and function of the heart. The heart, compared with other organs, has largely been insensitive to evolution indicating that the archetype of the original heart design in vertebrates was successful [27, 28]. Nevertheless, species-specific morphological adaptations, for example, bipedalism and change in the center of gravity, have occurred without alteration of the basic blueprint of the heart [28].

### Emergence of a circulatory system and the rise of cardiac biomechanics

The emergent tree from first eukaryotic cells starts 1.5 billion years ago [29, 30] and about 700 million years ago, an organized body plan emerged with radial symmetry (sponges, jellyfish) followed by creatures with bilateral symmetry and a dorsoventral axis. The first tubular heart appeared in the bilateralia which diverged into two branches: the Protostomes (invertebrates) and the Deuterostomes (vertebrates) [31]. Although impossible to prove (due to lack of fossilization), it is almost certain that contemporary mammals inherited their hearts from our common placental ancestors, originating at the end of the Mesozoic around 125 million years ago [32, 33].

Embryogenesis of the heart shows a common step consisting of a primary cardiac tube derived from mesodermal precursor cells converging in the midline [34] with peristaltic movements pushing fluid into the pericellular spaces without vessels [31]. However, the conversion to terrestrial activities increased body size and the appearance of endothermia (both mandating an increased metabolism) necessitated an altered circulatory system with closed pulmonary and systemic circuits. Small creatures thrive on diffusion through either an open or a closed system. An open system mandates high output with low pressure where the blood, lymph, and extracellular fluid are mixed together. There is a large circulating volume (20–50% of the body weight) with low velocity blood flow [35]. In a closed system, there is a higher pressure with lower circulating volume (6–8% of the body weight) where blood flows through arteries, veins, and capillaries with endothelial lining. This system is present in all cephalopods and vertebrates and shows multiple advantages with respect to an open system. The presence of blood as oxygen (O_2_) carrier increases the delivery of O_2_ by 20–40 times and this essential compound, some type of haem pigment, is present in almost all living beings [35].

**Fig. 1:**
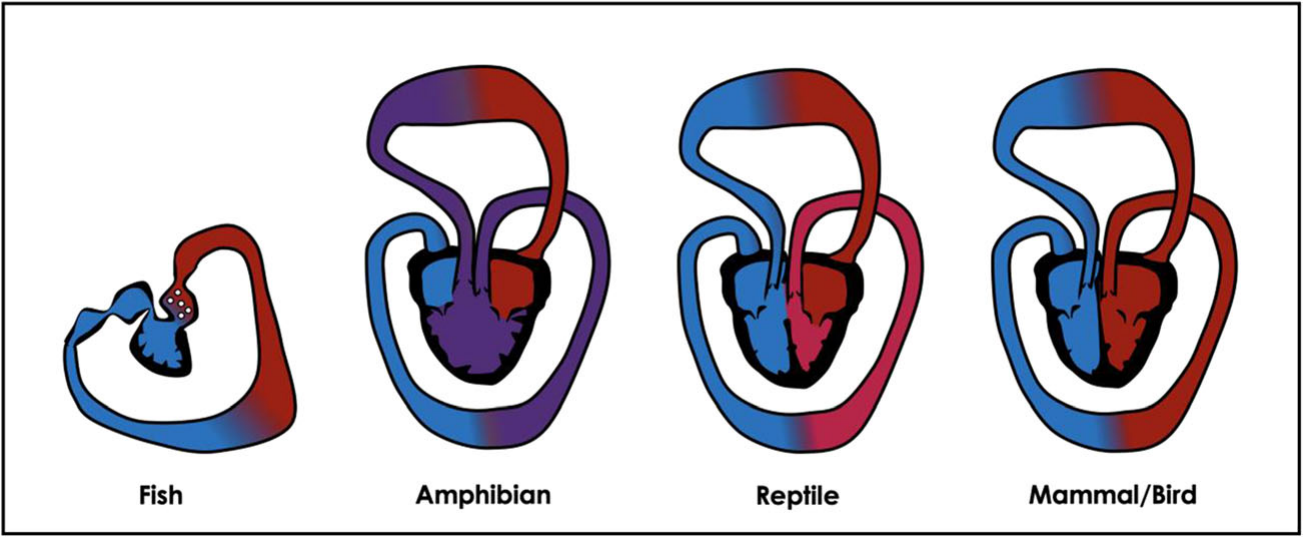
Interspecies cardiac anatomy. Timeline from a sequential pumping structure in fish to a univentricle in amphibian and 4 chambered structure with full septation in birds and mammals. Of importance is that birds and mammals independently evolved complete septation since the phylum of mammals took off from early terrestrial vertebrates (around 300 MYA), whereas birds are descendants from reptiles

All vertebrates stem from a common chordate ancestor, and cardiac tubes show sequential segments throughout the evolution of species very similar to the processes that occur during human embryogenesis [36] (Fig. 1). These primitive cardiac tubes consist of the sinus venosus with an atrium for inflow, atrioventricular canal, ventricle, and a conus arteriosus for outflow [27]. The heart modified its shape progressively among different classes of vertebrates [27, 29, 35], specifically: (1) progressive displacement of the inflow structures from caudal to dorsal (fishes) and cephalad (reptiles) position, (2) development of a high pressure left and low-pressure RV, (3) septation of the atrium in right and left cavity (amphibians), (4) septation starting at the interventricular groove separating the left from RV (crocodiles), (5) development of the RV from the proximal part of the conus arteriosus, and (6) disappearance of the sinus venosus (important for providing adequate preload when venous pressure is low (≤ 4 mmHg) in fish and amphibians) and conus arteriosus (which was placed downstream from the gills in fish to smoothen the systolic pulsatility) in birds and mammals (Fig. 1).

Another important change during evolution is the type of contractile fiber arrangement. Vertebrates have two types of arrangements: trabeculated, which is predominant in fishes and amphibians, and compact myocardium forming the entire ventricle of birds and mammals [35, 37, 38]. In trabeculated myocardium, there is a random distribution of fibers forming large trabeculations with the spongy appearance of the non-compacted myocardium (as seen in some CHD). Deep lacunae are present between these trabeculations necessary for the O_2_ supply through diffusion. This type of myocardium functions as an adequate pump, however, generating low pressure (≤ 25 mmHg) [39]. Trabeculated myocardium represents less than 50% of the ventricular wall in reptiles and has fully disappeared in birds and mammals. Compact myocardium is defined by well-organized fiber bundles that are able to generate high pressure but depend on the coronary vasculature for oxygenation. Consistent with their progressive ability to generate high pressures, compact myocardium forms 15–35% of the wall thickness in active fishes (like tuna), and most of the ventricle in reptiles, while the entire ventricles consist of compact myocardium in birds and mammals [38].

### Important species in the evolutionary tree and alteration of hemodynamics

Fish have a single circulatory tube with an elastic cone at the aortic root to cope with pulsatility (Windkessel effect) and a unidirectional atrioventricular and outflow valve to prevent backflow. The necessity for higher driving pressures by more active species determines the type of myocardium. More sedentary species have trabeculated myocardium, while more active species (tuna and shark) have compact myocardium with coronary arteries and pressures up to 80 mmHg [40].

In order to allow for adequate O_2_ exchange at the site of the lungs in species with higher metabolic needs, lower pressures in the pulmonary vascular bed are necessary to prevent edema (particularly in smaller mammals). Therefore, in birds and mammals, a complete separation of the pulmonary and systemic circulation is present [35, 41]. Separated circulations first occurred in octopi and are most pronounced in birds (birds have a higher systolic blood pressure than mammals: 150–170/70 mmHg) as high blood pressure allows for rapid transfer of O_2_ and nutrients and selective repartition of blood flow according to metabolic needs. Importantly, the process of complete septation evolved separately and independently in birds and mammals since the phylum of mammals took off from early terrestrial vertebrates (around 300 million years ago), whereas birds are descendants from reptiles [29, 36].

Another benefit of complete septation, besides less mixed blood and thus higher arterial O_2_ tension, is that it allows for thinner alveolar membranes and prevents interstitial fluid leakage. However, a major limitation of separate circulations is the fact that they are completely interdependent and necessitate, besides the reciprocal modification due to respiration of the stroke volume in each ventricle, a complex (auto)regulatory system. Another evolutionary tradeoff is the distance between the heart and the lungs; i.e., their proximity can be explained by basic physics describing that longer vessels cause a loss in pressure. It is important to note that the development of four chambers evolved out of necessity for a closed circuit and that septation occurred separating the same evolutionary ventricle into a right and left side. As such, cardiac embryological origins must have started to differ in both ventricles, however, difficult to determine when those patterns emerged through evolution (Fig. 1).

In summary, processes responsible for cardiac formation are based on very ancient cardiac lineages and within the first few weeks of fetal life, the human heart seems to repeat the evolution of the cardiac morphology which occurred in millions of years from worms to mammals.

### Embryology of the RV

The heart is the first organ to be formed during embryogenesis, starts beating on day 21, and is finished by 8 weeks after gestation. The heart formation consists of four key phases: tubular heart formation, cardiac looping, chamber formation, and complete septation with the development of the coronary arteries [42–45].

**Fig. 2:**
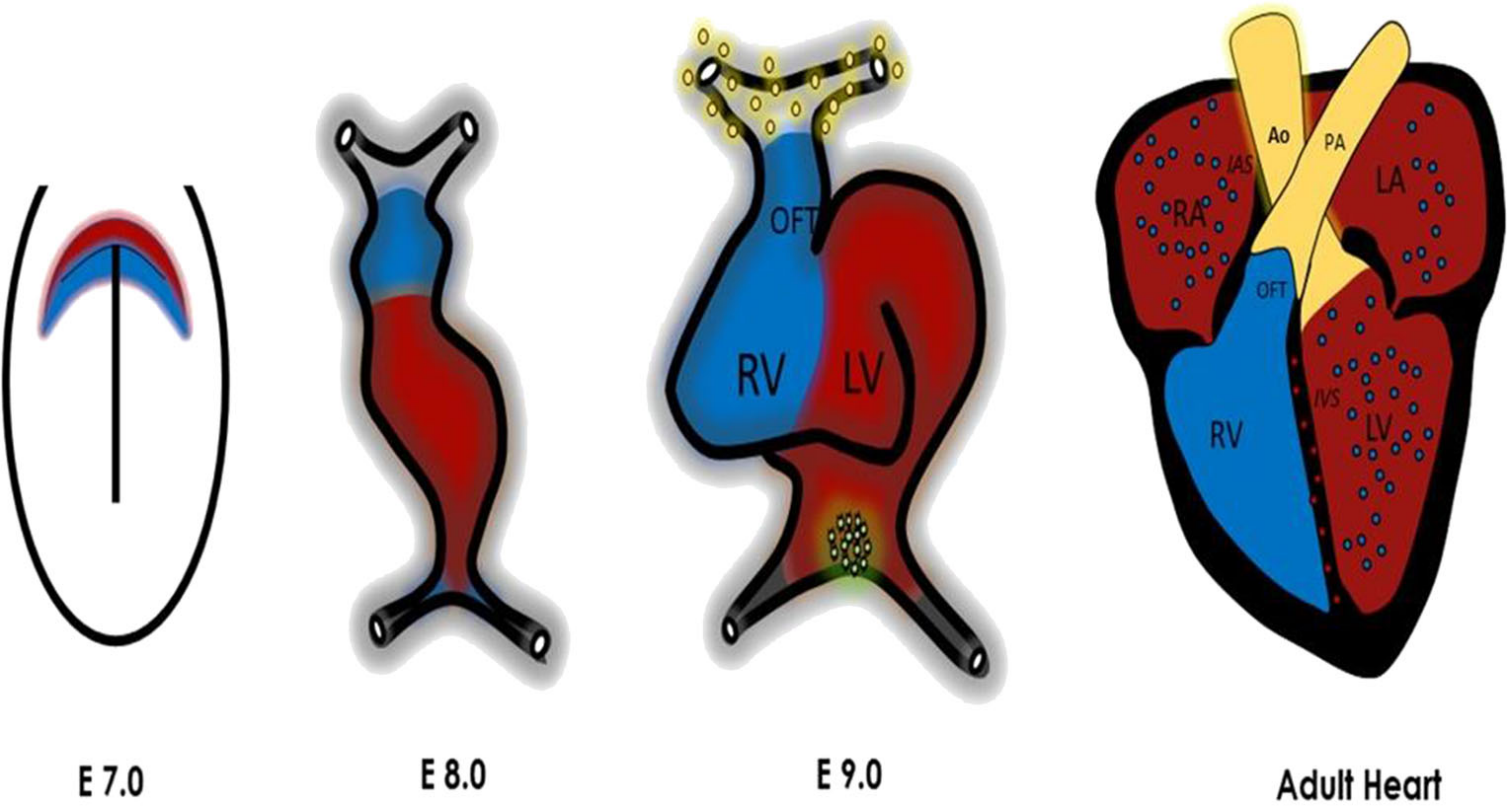
Summary of cardiac development in a mouse embryo. At stage E6.5, first cardiac development can be detected where the cardiac mesoderm is formed at the posterior side of the embryo (along the primitive streak). At E7, cells from the cardiac mesoderm migrate towards the anterior side of the embryo thereby forming two distinct progenitor pools called the first heart field (red) and secondary heart field (blue). The first heart field gives rise to the primitive heart tube which will eventually lead to the formation of the LV and parts of both atria. At E8, progenitors from the secondary heart field, which are located behind the primitive heart tube within the pharyngeal mesoderm (grey shade), migrate towards the primitive heart tube and will form the RV, parts of the outflow tract (OFT: which will later be the base of the aorta and pulmonary trunk) and also contribute to both atria. At E9.0, distinct poles at the inflow and outflow regions contribute to the formation of the epicardium, from the pro-epicardial organ (green), and the formation of smooth muscle cells within the aorta and pulmonary arteries, from the cardiac neural crest (yellow) respectively [38, 45]

RV myocardium derives from where cardiac progenitor cells meet the anterior heart field [44]. At first, the heart tube starts off as a flat sheet of mesodermal cells where cardiac progenitor cells coming from the anterior splanchnic mesoderm migrate to an anterior lateral position thus forming the bilateral heart primordia (primary heart field (PHF)) (Fig. 2) [42, 44, 45]. The cranio-caudal fusing of those paired heart primordia results in the formation of the primary heart tube which starts beating at day 21 [46]. The cranial regions of this heart tube become the ventricles while the caudal sections give rise to the atria. New research provided insight into cells derived from the pharyngeal mesoderm forming the secondary heart field (SHF) [45] (Fig. 2). In fact, the PHF gives rise to the LV, whereas the RV and proximal parts of the outflow tract are derived from the SHF [47]. The relevance of the SHF in cardiogenesis was presented by studies that labeled the secondary heart field by *Isl1* expression in Xenopus and zebrafish [48, 49]. Also, homozygous null mice for *Isl1* have no looping during cardiac formation where the outflow tract, RV, and a significant portion of the atria do not form [50]. It is currently unknown whether this different embryological origin affects altered loading conditions and possible maladaptation of the RV [16].

From this stage on, the heart loops forming a curl-like structure where the atria are connected and the left atrium is in continuity, through the atrioventricular connection, with the univentricle. This primitive ventricle connects to the bulbus cordis which will later form the RV and terminates in, what will be called, the truncus arteriosus [27, 42, 44]. Septation of the atria appears first at the beginning of week 5 and terminates by the end of that week. At the beginning of week 6, the interventricular septum forms, starting at the apex of the single ventricle [42, 44].

**Fig. 3:**
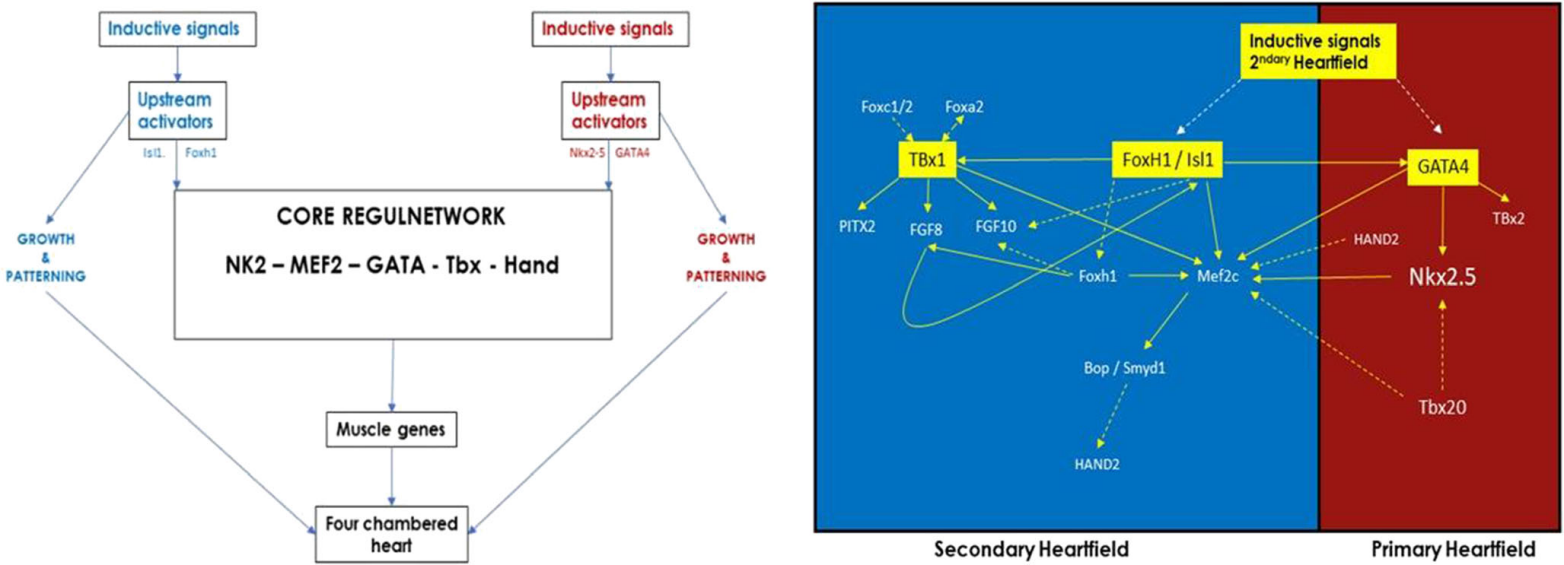
Inductive signals in primary and secondary heart field leading to the formation of a four chambered heart. Left panel: both upstream regulatory genes, *Isl1* and *Foxh1* for the SHF (blue) and *Nkx2-5* and *GATA4* for the PHF (red), activate genes in the core cardiac network (*NK2-MEF2-GATA-Tbx-Hand*). Right panel: regulatory interactions among transcription factors with intertwinement between the first and secondary heart field. Solid lines indicate direct transcriptional connections while dotted lines represent connections that are indirect or net yet shown to be direct. Adopted from [38, 51]

Cardiac cell lineages responsible for understanding cardiogenesis are of specific importance when interpreting congenital heart malformations but also in light of cardiac adaptations and failure (Fig. 3). Mesodermal induction, which is quintessential for heart development, is evolutionary conserved and regulated by a number of signaling pathways [52]. The mesoderm-derived PHF and SHF are the predominant sources for cardiomyocyte formation with only a minor contribution from the pro-epicardium [53]. In vivo and in vitro lineage tracings have shown that *Isl1+* marked SHF progenitor cells contribute to the formation of cardiomyocytes of the RV and both atria but also vascular smooth muscle cells and endothelial cells [51, 54]. Unlike progenitor cells form the SHF, PHF progenitor cells are marked by the ion-channel hyperpolarization-activated cyclic nucleotide-gated channel 4 (*Hcn4+*) expression and contribute to left ventricular and atrial cardiomyocytes [53]. Although still under debate, studies suggest that there is also a contribution to ventricular cardiomyocytes, arising from pre-epicardial cells undergoing epicardium-to-mesenchymal transition, migrating into the myocardium as epicardium derived cells [55, 56]. Interestingly, data from coculture experiments indicated that inductive signals originate from the anterior endoderm rather than from the cardiac mesoderm itself [57, 58]. Additional cardiogenic signals are derived from the organizer region [59] acting directly by inducing cardiogenesis in combination with anterior endodermal signals or indirectly through patterning of the early adjacent endoderm [60]. Earliest responses involve the induction of a core regulatory network which is activated through specific upstream activators from the first and secondary heart field where genes encoding factors of the *NK* homeodomain, *GATA*, *T-box*, and others have been found to exert functions of inductive signals during specification, patterning, and differentiation [60]. As presented in Fig. 3, it appears that most cardiogenic signal pathways (if not all) act in combination with tissue-specific transcriptional cofactors to exert inductive responses [60] reflecting an important expansion of ancestral regulatory genes throughout evolution and eventually cardiac complexity (reviewed in [61]). An explicit advantage of this region-specific control by a diversity of regulatory elements is that the evolutionary adaptation of specific cardiac structures is possible without affecting the whole organ [61, 62]. During the evolution of the human heart, complexity enhanced due to the co-option of different upstream inputs from the core regulatory network. It is this variation in upstream input that explains the development of the RV form the SHF despite the fact that both ventricles rely on the same transcription factors for the activation of the genetic program for cardiomyocyte differentiation [61]. One explicit transcription factor for the SHF is *ISl1*, however, not being cardiac specific. In order to activate downstream targets, such as the myocyte enhancer factor 2c (*Mef2c*) gene, it requires co-factors such as *GATA* and *Nkx2-5* expressed in both heart fields. A separate enhancer, *Foxh1*, acting downstream of *Isl1*, also activates *Mefc2* [63]. Additional genes, such as *Fgf8*, *Fgf10*, *PITX2*, and *Tbx1*, are also preferentially expressed in the anterior heart field where *Tbx1* has recently been shown to regulate the *Mef2c* gene expression [64].

It may be clear that no “master” regulatory gene is responsible for cardiogenesis and determining the evolutionary intertwinement of those core transcription factors proves to be challenging [61, 65]. Nevertheless, mutations in these core regulatory genes cause a spectrum of congenital heart defects such as atrial and ventricle septum defects and conduction abnormalities caused by mutations in *Nkx2-5* [66]. Deletions of a small piece of chromosome 22, more specifically 22q11.2, results in the DiGeorge syndrome with mutations in the *Tbx1* gene resulting in the tetralogy of Fallot due to failure of migration of neural crest cells to the heart. On the other hand, mutations in *Tbx5* are responsible for Holt-Oram syndrome [66]. Detailed descriptions of cardiac cell lineages are beyond the scope of this article and have been described in some excellent reviews [45, 61, 65].

To summarize, evolutionary pressure favored a closed system with a low resistance pulmonary bed allowing for thin alveoli to favor O_2_ uptake and a high resistance systemic vascular bed permitting local regulation of O_2_ delivery. This evolutionary design encompasses progressive septation of the heart, resulting in the building plans of the mammalian (and bird) heart. This blueprint was subsequently maintained across species, independent of lifestyle and habitat, where genetics from 3-chambered to 4-chambered hearts emerged from the same ancestor. Within the first weeks of fetal life, interspecies cardiac evolution is incorporated in embryogenesis where (1) in 20 days, a single tube (worm like) is present; (2) followed by a sequential pumping structure (fish like) with an atrium, ventricle, bulbus cordis, and truncus arteriosus at 28 days; (3) continuing into a two-chambered heart (like amphibians and reptiles at 30 days); and (4) eventually the heart gets fully septated (which is present in the bird, mammals, and crocodilians) by days 35–50. Cardiac regulatory genes have co-evolved during cardiac evolution with upstream signaling cascades that are intertwined, however, with specific genetic profiles for the RV (Fig. 3). One important factor in RV formation is *Isl1* which has been suggested as the key element of lineage diversification in the heart [67]. Despite the fact that these cells are not expressed in the RV and RV outflow tract, their function is necessary for the activation of SHF enhancers from both *Nkx2-5* and *Mef2c* genes [65]. It is clear that heart development is controlled by an evolutionarily conserved network of transcription factors which has been expanded and co-opted novel networks making it possible for the downstream alteration of specific cardiac regions, despite the common origin of cardiac precursors and inductive signals.

## Right ventricular form and function

### Morphology and microarchitecture

In the normal adult heart, the semicircular RV shape is lounged around the LV. The RV has a unique thin-walled triangular (in lateral section) and crescent (in cross section) shape with a sinus (inlet), apex and conus, or infundibulum (outlet), separated by the supraventricular crest. The shape of the RV is further determined by the interventricular septum with a concave shape towards the LV (under normal loading and electrical conditions) which is unaffected by the cardiac cycle. The thin-walled RV is anchored to the LV anteriorly and posteriorly both sharing the interventricular septum. The RV contains coarse trabeculations, a trabecula septomarginalis, providing innervation from the interventricular septum to the base of the anterior papillary muscle, and crista supraventricularis. The latter has a “U-shaped” morphology and is wedged between the tricuspid and pulmonary valve at the junction of the interventricular septum and the RV anterior wall and extends to the sub-pulmonary infundibulum.

**Fig. 4:**
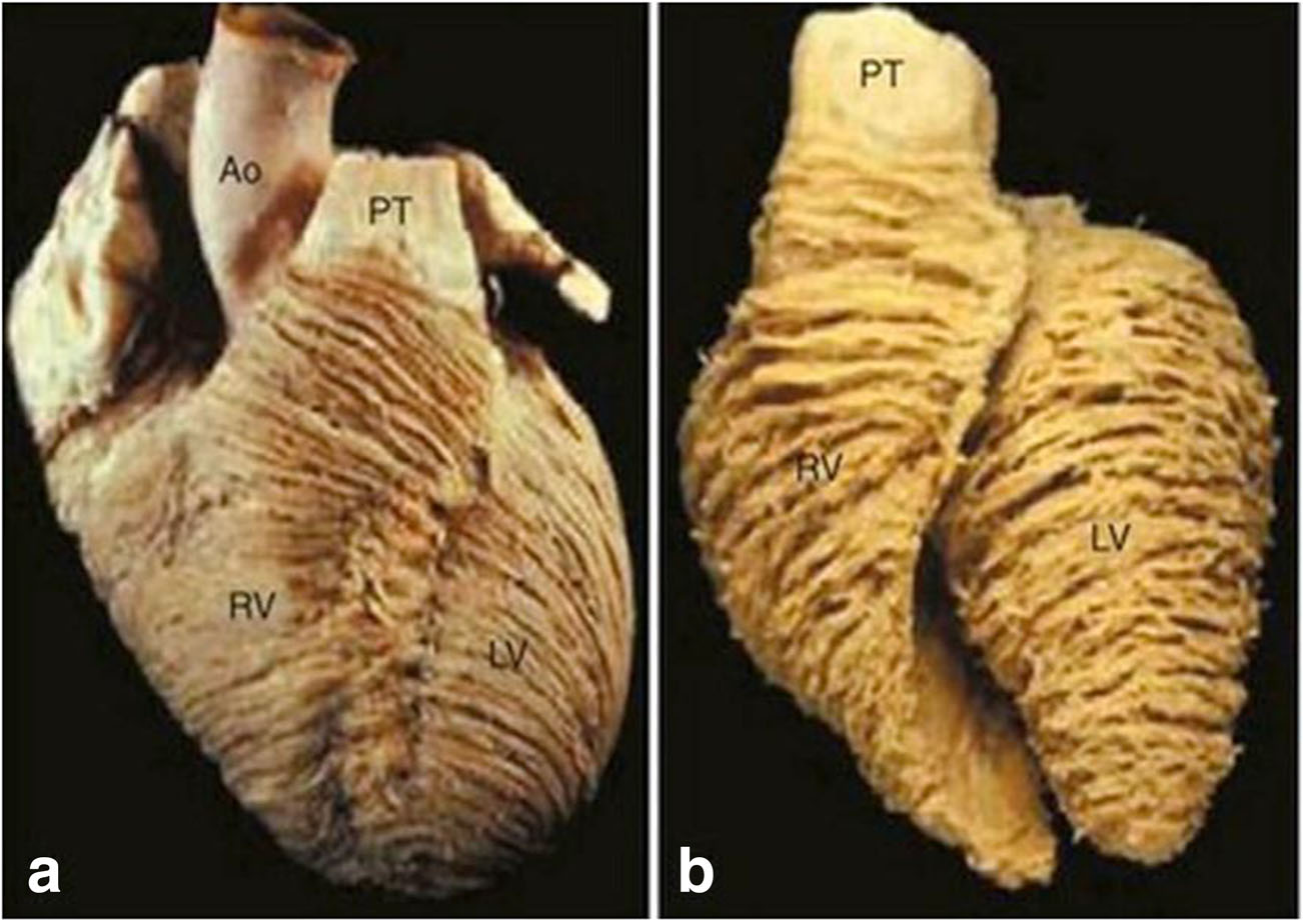
Microarchitecture of the normal cardiac ventricles. Panel **a** shows a normal heart with subepicardial fiber arrangement from circumferential to oblique. Panel **b** shows the looping of fibers in the deeper layer retaining a circumferential arrangement in the RV, however, changing from oblique to circumferential in the LV. Of importance is the interventricular groove where in the subepicardial layer, myofibers cross and are intertwined between ventricles. From Ho and Nihoyannopoulos [68] (with permission)

Microarchitectural alignment is chamber specific, where the RV, due to different loading conditions with a resultant specific force generation pattern (as discussed below), is composed of superficial and deep muscle layers. This alignment contributes to the complex contraction pattern of the RV including torsion (however almost non-existing compared to the LV), translation, rotation, and thickening [68, 69]. One additional difference in microarchitecture of the RV is the lack of a third circumferential layer, which is necessary for LV reduction in diameter due to higher pressures. The superficial RV layer has fibers with a circumferential orientation, parallel to the atrioventricular groove, that is angulated towards the endocardium and turn obliquely towards the RV apex before continuing into the superficial layer of the LV (Fig. 4). Furthermore, the deep musculature is longitudinally aligned from base to apex. In contrast, the LV has oblique fibers superficially, with circular fibers below, and longitudinally oriented fibers in the sub-endocardium [68]. This specific alignment creates a peristaltic wave from the sinus to the conus (duration of ~ 25–50 msec [3]) with a major radius of curvature of about 4 cm and a minor radius of curvature of around 0.8 cm [70] and is determined by two major mechanisms. First, a longitudinal shortening, which is the dominant movement of the RV, accounts for approximately 75% of RV contraction and presses the RV free wall against the septum. This creates a bellows effect [6, 15] and allows to generate an adequate stroke volume [68, 69, 71]. Second, this contraction is aided by free wall movement where horizontal fibers synergistically contract (as they pass the interventricular septum) with LV contraction thus emptying the RV into the low-resistance pulmonary circulation. Contrary to its more muscular counterpart, where a specific circular and looping muscular pattern is present, almost no torque (energetic rotation) is present to eject the blood.

Nevertheless, ventricular fiber orientation patterns of both ventricles are intertwined in a complex three-dimensional architecture defining their vectorial (functional) anatomy (Fig. 4), albeit with much controversy on the nature of this arrangement [72, 73]. Up-to-date, two theories exist defining the microarchitecture of the ventricles. On the one hand, there is evidence supporting a three-dimensional myocardial mesh defining a complex arrangement of aggregated cardiomyocytes with heterogeneous morphology where the specific orientation of the units into which they are aggregated, along with their specific loading conditions, dictates the efficacy of mural thickening [73–76]. On the other hand, much data supporting the myocardial band theory (of Torrent-Guasp) has been presented [77–80] where the helix is topographically divided into a basal loop and an apical loop running from the root of the pulmonary artery to the beginning of the central fold (i.e., the anterior papillary muscle) and from the central fold to the root of the aorta, respectively. The descendent fibers of the apical loop (after a 180 degrees turn at the central fold) are described to make a 90 degrees turn around the apex (of the LV) and become the ascendant fibers [81].

This specific morphological pattern translates into a specific RV function where volume handling, in respect to the pressure driven LV, is key. In such, in order to build up pressure in the LV, it is capable of creating a “torsion movement” which is created by opposing rotational movements of the base versus the apex. In contrast, the RV anatomical properties prohibit the production of torsion, even when facing extreme afterload [82].

### Biomechanical and hemodynamic profile

During fetal life, pulmonary resistance is high and blood is redirected creating a parallel circulation and the RV thus functions as a (low pressure) systemic ventricle. As a consequence, the RV is the dominant chamber in fetal life and accounts for about 60% of the cardiac output; hence, its wall thickness and force generation are equal to the LV [3]. Until the closure of the patent duct, the RV is exposed to systemic vascular resistance, which is more than pulmonary vascular resistance, as evidenced by regional blood flow analyses in fetal lambs [83]. Fetal cardiomyocytes display immature contractile features, featuring the β-myosin heavy chain (MHC) genetic phenotype characterized by lower adenosine triphosphate (ATP) activity and lower filament sliding velocity but with a higher ergonomic cross-bridge force generation than α-MHC [84, 85]. This may be an adaptation to the exposure of the RV to low partial O_2_ pressure with energy production being based on carbohydrates rather than fatty acids, thus creating glucose, lactate, and pyruvate, and has been suggested to be a feature of immature cardiomyocytes [5, 6]. Major signaling pathways that are activated in the fetal RV include hypoxia-inducible factor-alpha (*HIF-α*) and vascular endothelial growth factor (*VEGF*) that not only promote angiogenesis but are also associated with upregulated glycogenesis leading to good tolerance to hypoxia (which is better tolerated compared with the adult) [86–88]. Finally, fetal phenotype is associated with adrenergic receptors, calcineurin activity [16], and phosphodiesterase type 5 expression [89].

Immediately after birth, partial O_2_ pressure and shear stress in the pulmonary circulation increase, leading to the release of vasodilators (like prostacyclin and nitric oxide) and a decrease in secreted vasoconstrictors, such as endothelin-1. As a consequence, the pulmonary vascular resistance drops (and decreases even further later on), thereby decreasing pressures leading to an increase in compliance. Both the reduction in resistance and the increase in compliance contribute to the unloading of the RV [15], resulting in gradual progressive thinning of the RV wall [8]. Concomitantly, over the ensuing period of time, the patent ductus arteriosus closes creating a closed RV-pulmonary circulation circuit. While the RV becomes thin walled, LV mass increases as a result of altered physiology and bio-energetic demands as evidenced by molecular and genetic changes.

Compared with the fetal setting, there are several shifts in physiological processes. First, the metabolic pathway is adapted from glycolysis to fatty acid oxidation resulting in more ATP [90, 91]. Depending on the type of stress, there is a switch to glucose oxidation instead of fatty acid oxidation [5, 92, 93]. Also, due to changes in resistance in pulmonary and systemic beds, oxygen demands alter preferentially shifting regional blood flow to the LV free wall (as compared with the higher regional blood flow in utero of the RV free wall).

The pulmonary vascular bed has a low hydraulic impedance with high compliance. Therefore, the pressure generated by the RV is lower which, according to Laplace’s law, translates into the thickness of the RV free wall of about 3–5 mm (in adults) and a mass that is 1/3 to 1/6 of the LV [8] being 26 ± 5 g/m^2^ for the RV versus 87 ± 12 g/m^2^ for the LV [71]. Nevertheless, the RV has a 10–15% larger volume than the LV, of which the infundibulum accounts for approximately 20% of the end-diastolic volume in the normal RV [3]. This specific morphology represents much of the mathematical problems when analyzing and describing hemodynamics [94] compared with the more cylindrical LV and it is imperative to take into account that the ventricles not only share the pericardium, and are therefore confound to the same external forces, but also cardiomyocytes forming the LV and RV. It has even been estimated that roughly 30% of the contractile energy of the RV is generated by the LV [95].

In summary, biomechanics of the RV are complex and differ from the LV in that morphological configuration of the RV is based on a very specific physiological demand. It depends on the insertion of the fibers on the interventricular septum, intertwinement with fibers of the LV, the septum itself, the contraction of the LV, and resultant external forces from the pericardium. It may be clear that sole descriptions of RV contraction patterns (and LV contraction patterns for that matter) are futile and need to be addressed into a bigger multilayer vectorial three-dimensional picture. This being said, caution should be warranted when interpreting such results as it is difficult to match architecture with the existing morphological data [94] and clear definitions of the manner of cardiomyocyte aggregation within its boundaries are necessary [73] where future high-resolution morphological studies could possibly provide better understanding.

Differences between the right and left normal ventricular morphology and physiology are summarized in Table 1.

**Table 1:** Normal morphology and physiology of the RV versus LV. Adopted from [18]. *PVR*, pulmonary vascular resistance; CBF, coronary blood flow; *CBS*, coronary blood supply

	RV	LV
Formation		
Evolutionary development	Late	Early
Embryological origin	Secondary heart field	Primary heart field
Fetal characteristics	Thick RV wall (due to high PVR)	Thick LV wall
Postnatal characteristics	Drop in PVR + closure ductus arteriosus ➔ RV becomes thin walled	LV mass increases
Morphology		
Form	Thin walled with coarse trabeculations	Thick walled with thin trabeculations
	Triangular - crescent	Cylindrical
	Trabecula septomarginalis and crista supraventricularis	
Myocyte architecture	Predominant longitudinal orientation with angulated intrusion superficial myocytes towards endocardium	Predominant radial orientation in the mid layers with right-hand orientation subendocardial versus left-hand subepicardial
	Lack of circumferential layer ➔ abutment free wall against septum	Presence of a third layer of circumferential constrictor fibers necessary for the reduction in ventricular diameter due to higher LV pressures
Vascularization & CBF	Extensive collateral system and dual CBS, especially the first septal branch (from LAD) supplying the trabecula septomarginalis	Extensive collaterals
	Throughout the cardiac cycle	Predominantly during diastole
Physiology		
Resistance system	Low capacitance, low pressure pump	High resistance, high pressure pump
Cardiac output	Minimal isovolumetric periods with “hangout” period	Well-defined isovolumetric contraction and relaxation, no “hangout” period
	Faster twitch velocities	Lower twitch velocities
	Triangular/trapezoidal volume-pressure relationship	Square wave relationship
Energy expenditure	1/5th of the left	

## Pathobiology of remodeling, adaptation, and maladaptation of the RV

### Pressure versus volume overload

For many years, pathobiology of the RV was extrapolated from the LV and thus specific knowledge on the response of the RV on altered loading conditions is scarce, let alone, knowledge on increased volume loading [12–15]. Previous insights ascribed differences between both ventricles to the different structure and different loading conditions; however, as outlined above, those differences begin early on in embryogenesis exerted through specific molecular pathways [8, 18, 27]. Notably, RV failure is not an entity on itself but rather a continuum of clinical symptoms [96] related to severities of disease states in concurrence with previous definitions of HF [6, 97]. Despite the fact that RV failure can develop after ischemia, albeit regional or global, in the clinical setting, it is most often the result of increased afterload [15]. Studies have shown that even a slight acute increase in afterload can lead to profound decreases in RV stroke volume [98, 99]. This difference in ventricular response is particularly present in patients with CHD, more specifically in single ventricle physiology or a systemic RV physiology [19]. Many clinical trials already showed that standard therapy for LV failure does not improve RV failure in CHD [100–104]. Also, long-term survival analyses of single ventricle physiology showed faster progression to failure in the RV compared with the LV [19, 105, 106].

Another important group is represented by patients with longstanding PAH with HFpEF where eventually RV dysfunction develops in about one-third of all patients. This cannot solely be attributed to the afterload mismatch and identifies those patients with an increased risk of death [26, 107]. Moreover, the interventricular progression of the disease is apparent in patients with HFpEF constituting almost half of the patient group with HF [108]. Recent studies show that an important number of these patients display RV dysfunction which is associated with high morbidity and mortality [109, 110]. The group of Obokata presented for the first time that in these patients decline in RV function is coupled with adverse RV remodeling and dilatation [111]. Importantly, longitudinal changes in the right heart structure greatly exceeded the equivalent changes of the LV, and development of RV disease nearly doubled the risk of death in HFpEF [111]. Seemingly, the interventricular connection and function presents a pathophysiological continuum with chamber specific characteristics which we will discuss below.

Adaptations to chronic pressure overload, as evidenced by experimental PAH animal models [12, 16, 112–117], start with an acute phase RV dilatation which can be seen as the hallmark of RV failure [16, 17, 87, 118]. The associated increased wall stress consequentially triggers a compensatory RV hypertrophy and is essential in RV adaptation which has been shown to be a strong predictor for outcome in the LV, however, without a clear association in outcome for the RV [119]. Also, long-term studies of patients with, for example, single ventricle physiology show worse outcomes with earlier ventricular failure in systemic RV according to patients with a systemic LV [120]. The transition from adaptive to maladaptive biomechanics is clinically unpredictable and very poorly understood [5] and while many authors make strict divisions between them, recent advances tend to approach this transition as a continuum where accumulating changes in energy expenditure, biomechanics, and molecular pathways eventually tilt the balance towards maladaptation [5, 13]. Those transition phases may provide a good target for further possible interventions; however, minimal data exists.

Clearly, the design of the RV is not optimized for pressure generation, where the thinner wall and lower volume-to-surface-area ratio [3, 6] of the RV make it more compliant to increased preload, though, unable to cope with abrupt increases of pulmonary artery pressures. Every acute increase of preload or afterload instantaneously results in an increase in contractility proportional to its afterload trying to maintain stroke volume. When the increased contractility does not meet the arterial elastance, this ratio declines leading to ventricular-arterial uncoupling which can be seen as a sign of RV failure [12, 15, 121, 122]. This initial reaction normalizes after several minutes with adaptations resulting in increased contractility and normalization of end-diastolic volume [3] and is exerted through mechano-transduction and trophic paracrine signals from the stretched cardiomyocyte leading to changes in integrins, stretch-activated ion-channels and the major sarcomeric protein titin [3, 123, 124]. Interestingly, titin-based passive tension and stiffness are modulated by titin isoform transitions where during prenatal development, titin becomes stiffer (due to decreases in large (N2BA) to small (N2B) titin isoform ratio) [125] and during chronic HF, the isoform ratio can increase and lower titin-based passive tension [126].

Experimental volume loading studies show that RV contractility remains preserved for a long period, although contractile reserve may be compromised [127, 128]. Also, an important consequence of RV volume overload is LV dysfunction, evidenced by the reduced compliance and ejection fraction [3, 128] based on underfilling due to septal displacement and changes in LV geometry rather than a RV forward failure [3, 6]. Correspondingly, reorientation of RV myocardial fibers has been described, dependent on the underlying disease, to sustain RV ejection fraction and more importantly biventricular systolic contraction [129, 130]. Interestingly, patients with Eisenmenger syndrome are doing better and have a better outcome than those with idiopathic PAH possibly due to preconditioning from the prior volume loading or the regression into fetal genetic programming (see below) [6, 13]. However, caution should be warranted as adult survivors with Eisenmenger syndrome present a survival bias which may skew this outcome. The possibility to unload the RV is key where patients with PAH who received a pulmonary-to-systemic shunt (i.e., a Potts shunt)—creating a pop off for the RV—have improved outcomes [131] and, likewise, in the neonatal period, patients rarely die of advanced RV failure due to the open duct. Creating an unloading possibility, through atrioseptectomy, has recently gained more interest and has even been recommended in patients with HFpEF [132, 133]. The same holds true for adults with RV failure as a bridge to lung transplant [134] or as an additional therapy for PAH-associated RV failure where medical treatments fail [135, 136]. It is clear that the underlying mechanism is of importance and selected patients may benefit from unloading; however, the associated drop in systemic O_2_ tension should outweigh the increase in O_2_ delivery mediated by the increased cardiac output.

Overall, our understanding of RV response to chronic pressure overload, but also why certain patients are able to adapt and sustain very high pulmonary artery pressures, remains incomplete. In experimental models of RV failure, RV hypertrophy is present however not related to functional adaptation [12, 112, 137, 138]. As discussed by Borgdorff et al. [15], a caveat is present when interpreting experimental results because differences in loading conditions lead to different degrees of RV adaptations and hypertrophy and these studies may therefore not be compared directly [86, 112]. It is clear that, although some aspects of LV and RV cellular biology may be comparable, there are important differences in gene expression, mitochondrial function, energy production, production of reactive oxygen species (ROS), and angiogenic response to chronically increased workload. Responses to different loading conditions and pathophysiological bases for the development of RV maladaptation and eventually failure will be discussed below. Different adaptations of the RV versus the LV to altered loading conditions are presented in Table 2.

**Table 2:** The effects of altered loading conditions on the RV and LV. Adopted form [3, 18, 103, 139]. *ANP*, atrial natriuretic peptide; *DA*, dopamine receptor; *cAMP*, cyclic adenosine monophosphate; *Ca2+*, calcium ion; *MLCK*, myosin light chain kinase; *FABP*, fatty acid binding protein; *AngII*, angiotensin; *HIF*, hypoxia inducible factor; *ROS*, reactive oxygen species; *AO*, antioxidant; *SOD*, superoxide dismutase; *GPx*, glutathione peroxidase; *TNF-α*, tumor necrosis factor; *NADPH*, nicotinamide adenine dinucleotide phosphate

	RV	LV
Genetic and molecular pathways	HAND2 for RV formation	Expressed ANP
	GATA4 (mandatory for HAND2 expression) • Regulates *a*-myosin • Regulates gene for ANP ➔ not expressed in RVF	
	NKx2.5 ↑ (normally only present in fetal genetic program)	
Adrenergic receptors	↓*β*1, *α*1-, and DA1 receptors with ↓[cAMP] = ↓ inotropic response	↑ Contractility to a-1 adrenergic receptor agonists
	↓ Myofilament Ca^2+^ sensitivity through phosphorylation of MLCK (in eustress)	+ α1--signaling (in eustress) attributable to ↑ myofilament Ca2+ sensitivity
	Long-term norepinephrine infusion does not result in hypertrophy	Long-term norepinephrine infusion results in hypertrophy
Energy metabolism	Metabolic shift from fatty acid to glucose oxidation ➔ ↑ glycolysis • Already ↓ FABP in non-stressed state	↑FABP in non-stressed state
	Wnt pathway ↑ activation	Lesser activation Wnt pathway
	Inefficient energy metabolism	Improved energy metabolism
	Important pathways involved in glycolysis HIF-1 α and p38-MAPK	
	Chronic volume overload: early diastolic function with downregulation of regulators in ATP production pathway	
	↓ Mitochondrial complex I, III, and IV with ↓ resting mitochondrial membrane potential	↑ Resting mitochondrial membrane potential
Cellular matrix and fibrosis	AngII receptors (further?) uncoupled, with downregulation of Ang-II receptor subtype 1	AngII receptors uncoupled, with downregulation of Ang-II receptor subtype 1
	Fibrotic response to volume loading stronger	Weaker fibrotic response to volume loading
Vascularization and capillary rarefaction	↓ Capillary density with ↓ angiogenic response to hypertrophy (however, increased capillary formation has also been described)	
	Loss of coronary vasodilatory reserve	
	↑ Glycolysis in response to hypoxia, however, with ↓ energy production	↑ Glycolysis in response to hypoxia with sufficient energy production
Inflammation and oxidative stress	↑HIF-1*α* activation and complex II-mediated ROS production	
	AO enzymes (SOD and GPx) not activated with concurrent more ROS production (during hypertrophy)	↑AO enzymes (SOD and GPx) (during hypertrophy)
	Prominent role Nf-kB pathway in RVF	
	Macrophage infiltration	
	TNF-α upregulated only in RV	
	↑ IL1-*α* and IL-1*β*	↑ IL1-*α* and IL-1*β*
	NADPH oxidase and mitochondrial complex II as primary source for ROS	NADPH oxidase as primary source for ROS

#### Genetic alterations and molecular pathways

Genetic cardiac morphogenetic analyses already showed a different embryological origin (as discussed above and shown in Fig. 3) where *HAND2* expression is necessary for RV formation. In such, the transcription factor *Bop* is known to be a transcriptional target of *Mef2c* and *GATA4* which are mandatory for *HAND2* expression. Also, *GATA4* regulates gene expression of α-MHC in the cardiac muscle and the gene encoding for atrial natriuretic peptide [17, 19, 87, 140]. With increased afterload of the RV, gene expression of α-MHC isoform declines, and the slower but energetically more favorable β-MHC is upregulated recapitulating the fetal gene pattern [12, 15, 89, 112, 141]. As reported by Guihare et al., ventricular-arterial uncoupling was strongly associated with the upregulation of β-MHC expression and suggested that dynamic changes in myosin expression may determine RV work efficiency [5, 142] which is correlated with a metabolic shift from fatty acid to glucose oxidation [5]. Interestingly, the change in α-MHC to β-MHC ratio is not related to the degree of RV dysfunction and the question arises whether the switch to a fetal program is an adaptive response to cope with adverse remodeling or whether the switch is rather protective in the decompensating heart. The switch to fetal programs has been addressed in some excellent reviews [141, 143] and years ago, the group of Thompson [144] already established that the transcription factor *Nkx2-5* is upregulated which is normally only present in the fetal heart. However, up-to-date, the precise function of this factor and its pathways are incompletely understood [145].

Nevertheless, individual genetic differences play a major role as some patients, with the same degree of afterload, develop more RV hypertrophy than others. Small studies have shown that patients with a non-DD angiotensin-converting enzyme polymorphism had elevated right atrial pressures and decreased cardiac output [146] suggesting that genetics play a part in controlling RV hypertrophy [6, 147]. The hypertrophic response is induced by several signal pathways (i.e., the calcineurin-*NFAT* pathway) and resembles that of the LV [15, 16]. However, recent analyses showed that there are important differences in expression of regulatory genes *C-fos*-induced growth factor (*FIGF*), transport protein particle (*TRAPPAC*), and connective tissue growth factor (*CTGF*) in the non-failing RV compared with the LV. But also, in the failing RV, fibrillin 2 (*FBN2*), *CTGF*, *SPARC*-related modular calcium binding 2 (*SMOC2*), and *TRAPP6AC* were differentially expressed with respect to the LV [140]. *FIGF* protein is a member of the platelet- and vascular-derived growth factor which is active in hypoxia-induced angiogenesis, lymphangiogenesis, and endothelial cell growth. In infarcted models, *FIGF* acts as a profibrogenic mediator stimulating myofibroblast growth and type I collagen synthesis [148] representing the possibility that, if *FIGF* expression is reduced, physiological remodeling may be impaired [140]. *CTGF* (also known as *CCN2*) is a multicellular protein of the family of extracellular matrix-associated heparin-binding proteins and has important roles in cell adhesion, migration, proliferation, and angiogenesis [149]. Interestingly, *CTGF* is important in normal embryological development, however, has been linked to adult pathophysiology of, for example, atherosclerosis and fibrosis [150]. Despite varying results reported on the association between *CTGF* levels and RV function, the recent findings of Williams et al. suggest that the role of *CTGF* in fibrosis and cardiomyopathy may be increased in right heart disease and RV failure [140].

As stated, the downregulation of FIGF and upregulation of *CTGF* may indicate that there are specific differences in response to stress between the LV and RV where inflammation plays a major part [140]. Specific implications of *TRAPPAC/TRAPP6AC*, *SMOC2* (which is active in vascular smooth muscle proliferation and migration), and *FBN2* (which functions as part of the extracellular matrix in connective tissue disease) in cardiovascular disease need to be elucidated.

#### Cellular matrix and development of fibrosis

The extracellular matrix consists predominantly of collagen with relatively small amounts of laminectin, fibronectin, and elastin, and is able to influence the contractile function and morphological configuration of the heart [151–153]. Matrix homeostasis is tightly coupled with a myocardial function where increased collagen content (e.g., fibrosis) is strongly linked to the transforming growth factor (*TGF)-β* and degradation of the matrix by metalloproteinases [151, 153–156]. RV fibrosis in response to increased afterload has been shown in several studies [112, 121, 138]; however, much variation exists in the amount of fibrosis seen in different animal models and time to develop [15]. Moreover, studies evaluating cardiac fibrosis in PAH and chronic thromboembolic PAH show that fibrosis develops in the free wall, the ventricular insertion points, and in the septum (reviewed in [156]). However, contrary to the assessment of the insertion points, the free wall has received less attention due to technical difficulties related to the thin-walled RV [156]. As presented by the group of Andersen, this may create a bias leading to the assumption there is an absence of RV fibrosis in PAH where fibrosis should be seen as a dynamic process and established fibrosis is (partially) reversible [156].

Since fibrosis is closely coupled to end-systolic elastance, it would be interesting to try and alter the fibrotic state; nevertheless, more recently, a study by Borgdorff et al. showed no relationship between the amount of fibrosis and the degree of myocardial dysfunction (as corroborated by the group of Crnkovic et al. [157]). As stated by this group, it would be reasonable to see fibrosis of the RV as a consequence of the adapting and possibly failing RV in response to altered loading condition rather than to implement it in the pathophysiological pathway leading to RV failure [15, 112]. If fibrosis develops, however, it seems to be much less than in the pressure-overloaded LV possibly explaining why RV function recovers in patients after lung transplantation [139]. Studies trying to reduce fibrosis, by using beta-blockade [121], ROS scavengers [116, 158], and prostacyclin [159], also lowered afterload and it is thus impossible to link the effects to a direct interaction on fibrosis [15]. Also, therapeutic strategies targeting fibrosis in LV failure did not improve RV function in RV failure in an experimental setting [160] implying that characteristics of fibrosis are “chamber specific” and further research should explore these differences between the LV and RV.

An important factor correlated to cardiomyocyte hypertrophy, contractile dysfunction, and fibrosis in angiotensin II (AngII). The group of Rouleau already showed that AngII-receptors are uncoupled in a rabbit RV hypertrophic model leading to increased levels of AngII with adverse contractility which could be effectively reversed by Ramipril [161]. Also, fibrosis, which is secondary to inhibition of collagen degradation and extracellular matrix inflammation [17], can be altered with the administration of ACE-inhibitors and AngII type 1 receptor blockers [162, 163]. Nevertheless, Borgdorff et al. showed that targeting fibrosis did not affect RV adaptation [160]. Also, the group of van der Bom presented that Valsartan did not affect RV function in the systemic RV [103]. The only drug therapy that showed positive effects in experimental strategies has been beta-blockade [164]. From these observations, trials have shown that the administration of beta-blockade in patients with PAH is safe; however, the efficacy is yet unknown [165]. Furthermore, for pediatric HF patients, beta-blockade has not been shown to be effective and a sub-group analysis in patients with CHD even showed possible adverse outcomes (the study was underpowered) [100].

In summary, cardiomyocyte dysfunction is not the sole factor to transform the hypertrophic RV into a failing one. Chronic overload of the RV leads to extracellular matrix changes which are prominent in patients with PAH; however, fibrosis was minimal in compensated RV hypertrophy [17, 153, 166] and the question is raised if fibrosis should be discussed as part of the pathophysiological pathway.

#### Blood flow, energy metabolism, and oxidative stress

The right-sided blood supply differs from the left, as it is dependent on the dominance of the coronary system. In 80–85% of the patients, there is a right dominant coronary system supplying most of the RV, that is, the margo acuta by the marginal branches and the posterior wall and inferoseptal region by the posterior interventricular artery. The anterior wall and anteroseptal region of the RV are supplied by the anterior interventricular artery. Given the lower workload of the RV, O_2_ demand is lower than that of the LV in normal physiology. Also, due to lower pressures in the unstressed RV, coronary blood flow occurs throughout the complete cardiac cycle [15, 167]. Together with the more extensive collateral system (dual delivery), with a major branch of the first septal artery supplying the trabecula septomarginalis, and a more adaptable possibility to extract O_2_, the RV is relatively much more resistant to irreversible ischemia than is the LV [168]. However, when afterload increases with increased O_2_ demand, coronary perfusion occurs mainly in diastole [169] leading to a continuous O_2_ supply-demand imbalance [15]. Also, experimental studies on PAH showed decreased capillary density [121, 138, 159], without beneficial hemodynamic changes when administrating prostacyclin [159], suggesting a decreased coronary vasodilatory reserve. Moreover, the loss of vasodilatory reserve could be involved in the decline of RV function and the involvement of insufficient blood supply to the RV has been supported by Bogaard et al. who showed that chronic pressure overload is not sufficient enough to induce RV failure [17]. Nonetheless, the transition from RV adaptation to RV failure is defined by the occurrence of capillary rarefaction [138] which is a decrease in the number of perfused capillaries in an area of tissue, with decreased expression of VEGF in chronic RV dysfunction [170]. Up-to-date, however, it is still unclear whether RV ischemia is implemented in the transition from the compensated to the failing RV and conflicting reports exist on the role and extent of capillary rarefaction in RV failure. Recent research indicates that early RV remodeling in PAH is associated with RV angiogenesis and preserved RV function [171] while RV maladaptive response to chronic pressure overload is presented as a mismatch between angiogenesis and workload [172]. Furthermore, the same group recently showed that myocyte capillary rarefaction may be a sign of end-stage RV failure, irrespective of the etiology of PAH [173]. Another aspect that needs to be accounted for in the maladaptation of the RV is the modification of the cardiomyocytes. The group of Graham showed that cardiomyocyte modification and decreased substrate delivery is a major driver for RV maladaptation rather than capillary rarefaction [174] and can possibly explain the rapid recovery in RV function after bilateral lung transplantation for PAH. This recovery is too rapid to be explained by changes in RV structure and maybe more consistent with the reversal of intrinsic cardiomyocyte pathologies [174, 175].

Despite the difference in workload in the non-stressed state, energetic profiles of both ventricles are largely the same in terms of glycolysis, tricarboxylic acid cycle, oxidative phosphorylation, cellular aerobic capacity, and volume fraction of mitochondria [19]. However, the RV displays a slightly lower expression of fatty acid-binding protein [176]. Under normal conditions, the adult heart thrives on fatty acids for biosynthesis and energy production [19]. Nevertheless, under stress (i.e., with increased afterload with the onset of hypertrophy), a switch towards glucose and lactate occurs and glucose metabolism shifts from complete oxidation through the Krebs cycle towards glycolysis solely, yielding less ATP but using less O_2_ per ATP [15]. These alterations may be beneficial during acute stress, however, inadequate for longer maintenance of cardiac metabolism [15, 19, 91]. In respect to volume overload, the few studies that have been performed show an early diastolic dysfunction with preserved systolic function [14] and downregulation of regulators in the ATP production pathway (phosphofructokinase and aconitase) [5, 6, 19]. Nevertheless, studies illustrate that inhibiting fatty acid oxidation through trimetazidine increased cardiac output in a model of mild RV dysfunction due to pulmonary arterial banding [177] and in patients with diabetic cardiomyopathy [178]. The decreased glucose oxidation in the overloaded heart is related to the activation of pyruvate dehydrogenase kinases, which inhibits pyruvate dehydrogenase, thus preventing pyruvate from entering the Krebs cycle and increasing reliance on glycolysis for ATP production [19]. Inhibition of pyruvate dehydrogenase kinases by dichloroacetate has been shown to improve glucose oxidation and RV repolarization, thereby restoring RV function in rats with pulmonary PAH [179]. However, as noted by Borgdorff et al., the concomitant reduction in RV systolic pressure in a fixed afterload (due to banding) complicates the interpretation of these results [15].

Although several studies have reported either increased uptake of labeled glucose analog [180] or increased expression of glycolysis related genes [177, 179, 181–183], most of those studies were performed in rats without clinical manifestations of RV failure [15]. No data exists on alterations in substrate utilization (specific in CHD) and further experimental studies are needed in models displaying more severe RV failure. Furthermore, as described in solid tumors [184], chronic pressure overload in the RV also results in suppression of the mitochondrial oxidative function [142, 181]. However, mitochondrial structural changes are not well correlated with the severity of RV dysfunction [17, 142]. Important pathways involved in glycolysis and RV failure are *HIF-1α* and *p38-MAPK* [19]. Also, *HIF-1α* activation is associated with mitochondrial complex II-mediated reactive oxygen (ROS) production [19, 185] as evidenced in monocrotaline-induced hypertensive rats [116]. Furthermore, the increased production of free radicals could in part be counteracted by scavengers in PAH [116, 186]. Nevertheless, the evidence is present that hibernating mitochondrial metabolism is involved in the transition from RV hypertrophy to maladaptation [5, 142]. Recently, the group of Zuurbier used oxygen-dependent quenching of mitochondrial O_2_ tension to show that in rats with PAH, during the transition to RV failure, there is downregulation of in vivo mitochondrial O_2_ consumption in the absence of hypoxia [187]. At rest, both RV and LV exhibit the same mitochondrial profiles that only diverge when subjected to increased afterload [19]. Interestingly, when analyzing ROS production in both ventricles, major differences appear. First, when assessing mitochondrial function through its membrane potential, the resting RV shows a lower potential compared with the LV but increases with RV hypertrophy and is mediated through *NF-kB* [19, 188]. Next, upon increased loading conditions, oxidative stress increases earlier (with an early failing of antioxidant systems) and to a greater extent in the RV as compared with the LV where the redox state remains balanced even at a more advanced stage of failure [19, 189, 190]. In fact, the antioxidant activation pattern in a pressure-overloaded rat model displayed that superoxide dismutase and glutathione peroxidase are not activated at all in the compensated stage [191], predisposing the RV to ROS induced damage at an earlier stage [19]. Furthermore, there also seems to be a difference in ROS sources for both ventricles. In the LV, nicotinamide adenine dinucleotide phosphate (NADPH) is the primary source of ROS generation while in the RV, both NADPH oxidase and mitochondrial complex II-derived ROS are increased which would suggest greater importance for mitochondrial ROS production in RV failure [19, 116].

This progression from eustress to distress is associated with systemic activation of pro-inflammatory cytokines which accelerates the progression of HF [192]. Such an inflammatory state forms the connection between pressure load and apoptosis and is based on mechanical damage, oxidative stress, and neurohumeral signaling [15]. The group of Testa previously showed that increased levels of circulating cytokines are associated with the NYHA functional class in HF patients [193]. However, up-to-date, it is still unclear whether inflammation forms the basis for the transition from RV adaptation to failure. More specific, as PAH has a multifactorial nature, it is difficult to isolate an initial trigger of RV inflammation. Nonetheless, local stressors and systemic (neurohumeral) cascades could promote a “pro-inflammatory” environment in the overloaded RV [192]. Several studies support this general idea, although based on different experimental models of pressure-induced RV failure. By using a plant-derived alkaloid (MCT) to induce pulmonary EC injury [194], the tumor necrosis factor *(TNF)* superfamily appears to be implemented in the pathogenesis of RV failure through the *Nf-kB* pathway [195] in progressive MCT-induced PAH, albeit not in the stable form [196]. Also, in studies using pulmonary artery banding, several chemokines were upregulated with isolated (to the RV) increased leukocyte infiltration [197] and a prominent role for the *Nf-kB* pathway in RV failure [198]. Furthermore, the group of Guihare recently addressed the issue of macrophage infiltration in a rat RV dysfunction model strongly correlating with RV systolic dysfunction [199]. However, the exact role of macrophage infiltration needs further elaboration [153]. Interestingly, when creating a left-to-right shunt-induced PAH model, *TNF-α* and pro-inflammatory cytokines were also upregulated [200]; however, only *TNF-α* was solely upregulated in the RV while *IL1-a* and *IL-1b* concentrations were increased in both ventricles indicating a progression towards biventricular failure. Whatever the experimental setting may be, it is clear that inflammatory pathways are more commonly dysregulated in RV tissue in both failing and non-failing phenotypes [140].

Overall, a large amount of studies already showed that cellular changes in ventricular remodeling are stimulated by neurohormones (e.g., catecholamines and AngII), inflammatory cytokines, and wall stress which in part are mediated and exacerbated by the oxidative/nitrosative pathway [201]. Based on the different explanations on the transition from RV adaptation to RV failure, it is feasible to assume normal ROS physiology is hampered [202] where the source of ROS production has been a topic of debate. Several studies have indicated xanthine oxidase [203, 204], uncoupled endothelial nitric oxide synthase [205, 206], NADPH oxidase [203, 207], and mitochondria [116, 208] as the primal sources of ROS. However, a discrepancy between the experimental proof of the source and its clinical value exists. For example, uric acid (which is the product of xanthine oxidoreductase) is increased in the failing heart [203, 209] and related to a poor outcome [210]. Nevertheless, trying to inhibit xanthine oxidase showed no real improvement in symptomatic HF patients [211]. More recently, NADPH oxidases have been suggested as a consistent source for concomitant ROS production, more specifically by activation of the catalytic subunit *gp91*^*phox*^ and GTP-binding protein *Rac1* [116, 212]. Another major source comes from the mitochondrial apparatus [116], though keeping in mind that the produced superoxide itself can result in further production of ROS downstream. This initiates a downwards spiral with, for example, uncoupling of endothelial nitric oxide synthase, thereby producing superoxide instead of nitric oxide [201, 206].

## Conclusion

For many years, the RV was deemed to be a lesser contributor to the contractile function of the heart, mainly based on early experiments suggesting that the RV functions as a passive conduit and is subordinate to the LV. Since the Working Group Statement in 2006, progress has been made in understanding the pathophysiological and pathobiological mechanisms of RV failure due to abnormal loading conditions; however, many inconsistencies exist in the literature contributing to the present knowledge hiatus.

It is important to note that the development of four chambers evolved out of necessity for closed circuits with very different mechanical properties and differences between the left and right heart components have been embedded in the genetic profile. Indeed, multiple studies showed significant differences between the RV and LV on a morphological, physiological, and molecular level, resulting in a different adaptation to altered loading conditions and providing a potential explanation for the dissimilar responses of the ventricles to current HF therapies.

RV adaptation to increased afterload is characterized by dilatation, increased contractility, and hypertrophy, whereas the progression to RV failure is characterized by a progressive decline of diastolic function and, despite increased contractility, a disturbed ventricular-arterial coupling. An important part is the inefficient energy metabolism, with a metabolic switch from fatty acid to glucose utilization, which is apparent in RV adaptation; however, its role is still unclear in RV failure. Also, despite its maintained coronary blood flow throughout the cardiac cycle, the diseased RV possibly lacks the necessary increase in capillary density with a loss of coronary vasodilatory reserve. Nevertheless, the role of capillary rarefaction remains controversial and a distinct modification in cardiomyocytes exists with a decreased substrate delivery for a certain amount of workload. This hypoxic environment possibly aids the progression towards RV failure with a lack of antioxidant enzymatic response and amplifying the redox imbalance.

It seems that we are only now at the beginning of our understanding of the (patho)biology of RV remodeling and it is crucial to define specific molecular pathways responsible for this downwards spiral. Better yet, defining precise molecular mechanisms that elucidate the higher susceptibility of CHD patients to progress from a compensated state to overt RV failure would provide a concrete base for the development of “chamber-specific” HF therapies. It may be clear that there is an intertwined ventricular functional continuum within the process of HF and, based on the topics presented in this review, it is our understanding that RV failure forms a separate entity within this spectrum. In such, there should be a theoretical “point of no return” within this continuum of RV maladaptation to failure where our developmental blueprint plays an important role. Future research should therefore include comparative anatomical, developmental biological, and biomechanical approaches in the search for RV specific therapies.

### Key points

• Specific coupling between form and function in the right ventricle

• Evolutionary proxies are present in camber formation

• Specific molecular entities determine right ventricular biomechanics

`• Multifactorial approach necessary when describing the right ventricular failure
